# Properties and Clinical Application of Three Types of Dental Glass-Ceramics and Ceramics for CAD-CAM Technologies

**DOI:** 10.3390/ma3063700

**Published:** 2010-06-19

**Authors:** Christian Ritzberger, Elke Apel, Wolfram Höland, Arnd Peschke, Volker M. Rheinberger

**Affiliations:** Ivoclar Vivadent AG, Bendererstr. 2, LI-9494 Schaan, Principality of Liechtenstein; E-Mails: elke.apel@ivoclarvivadent.com (E.A.); wolfram.hoeland@ivoclarvivadent.com (W.H.); arnd.peschke@ivoclarvivadent.com (A.P.); volker.rheinberger@ivoclarvivadent.com (V.M.R.)

**Keywords:** biomaterials, glass-ceramics, ceramics, clinical applications, metal-free, CAD/CAM, dentistry

## Abstract

The main properties (mechanical, thermal and chemical) and clinical application for dental restoration are demonstrated for three types of glass-ceramics and sintered polycrystalline ceramic produced by Ivoclar Vivadent AG. Two types of glass-ceramics are derived from the leucite-type and the lithium disilicate-type. The third type of dental materials represents a ZrO_2_ ceramic. CAD/CAM technology is a procedure to manufacture dental ceramic restoration. Leucite-type glass-ceramics demonstrate high translucency, preferable optical/mechanical properties and an application as dental inlays, onlays and crowns. Based on an improvement of the mechanical parameters, specially the strength and toughness, the lithium disilicate glass-ceramics are used as crowns; applying a procedure to machine an intermediate product and producing the final glass-ceramic by an additional heat treatment. Small dental bridges of lithium disilicate glass-ceramic were fabricated using a molding technology. ZrO_2_ ceramics show high toughness and strength and were veneered with fluoroapatite glass-ceramic. Machining is possible with a porous intermediate product.

## 1. Introduction

From the eighteenth to the end of the nineteenth century, dental restorations were individually crafted. The eighteenth century saw the introduction of feldspathic materials, which were used for this purpose [1,2]. This type of ceramic was used for jacket crowns and crowns in the anterior region [3,4]. Subsequently, these materials were further developed. Also a new system was introduced in dentistry that uses metal to increase the strength of the porcelain to produce feldspathic ceramic fused to metal copings [5,6,7,8]. To this day, these PFM (porcelain-fused-to-metal) materials are being used very successfully.

At the end of the twentieth century, the development of all-ceramic solutions was strongly promoted. In response to the rising demand for highly esthetic products, glass-ceramics and polycrystalline sintered ceramics were developed to satisfy the clinical requirements of dentists as well as the esthetic expectations of patients.

Nowadays, not only the clinical and medical aspects of the treatment are of importance. The demands of patients for an attractive solution also have to be met, because today’s patients expect dental restorations to imitate the optical properties of natural teeth. Concurrently to the development of metal-free restoration techniques, ways of shortening the treatment time for patients as well as the manufacturing time of restorations were explored. In the process, the press technique (molding technology) was developed [9,10], which has firmly established itself in dental laboratories, since it produces restorations of a very high standard. In addition, CAD/CAM methods have become increasingly popular in the dental world [11,12,13]. These processing techniques, which are well-known from manufacturing systems engineering, have been adapted to meet dental requirements in recent years. As a result, this machining technology has become indispensible in the fabrication of dental restorations.

CAD/CAM procedures have not only been used in dental laboratories (lab-side), but also in dental clinics (chair-side). Due to the rapid entry of this machining technology in dentistry, new ceramic materials such as biomaterials for dental restoration have had to be developed which satisfy the requirements of dentists and their patients.

These requirements are listed below:

Requirements of the dentist and patient:
  ○ High strength and toughness depending on the required indication
  ○ High durability as dental restorative material
  ○ Excellent optical appearance (translucency, brightness, color and fluorescence like that of natural teeth)
  ○ Easy handling (no additional extensive treatment after the CAD/CAM process)
  ○ Easy placement of the restoration on natural dentin
Requirements of CAD/CAM technology:
  ○ No chipping
  ○ Easy processing
  ○ Preferable: small apparatus for dental clinics

In this paper, three different types of material systems, which have been developed specially for CAD/CAM processing, are described. In clinical situations, the choice of material used to fabricate a lasting, highly esthetic restoration is dictated by the pre-operative situation of the patient. The machining procedures with CAD/CAM methods are presented on the basis of most relevant clinical cases.

## 2. Materials Systems

### 2.1. Type I: Leucite-based glass-ceramics

**Figure 1:**
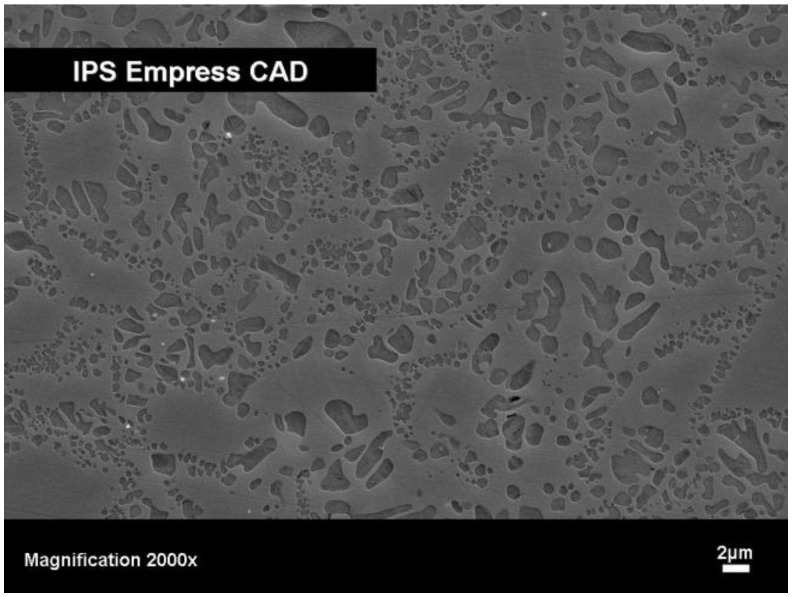
SEM image of a leucite-type glass-ceramic (IPS Empress^®^ CAD, Ivoclar Vivadent AG), fracture surface etched with 3% HF for 10 seconds.

Glass-ceramics based on leucite, K[AlSi_2_O_6_], show exceptional biocompatibility. Apart from their good chemical, physical and mechanical properties (Table 1), this type of glass-ceramic is well suited for computer aided machining. This type of glass-ceramic is produced by a method in which the nucleation and crystallization of a base glass is controlled [14]. The base glass is composed in the K_2_O-Al_2_O_3_-SiO_2_ system and important additives that influence both nucleation and crystallization. As a result of the controlled surface activation of the base glass by fine grinding and subsequently heat treating it, leucite crystals are precipitated. The final product shows a crystal content of 35 to 45 vol % with a crystallite size of 1-5 μm (Figure 1).

**Figure 2:**
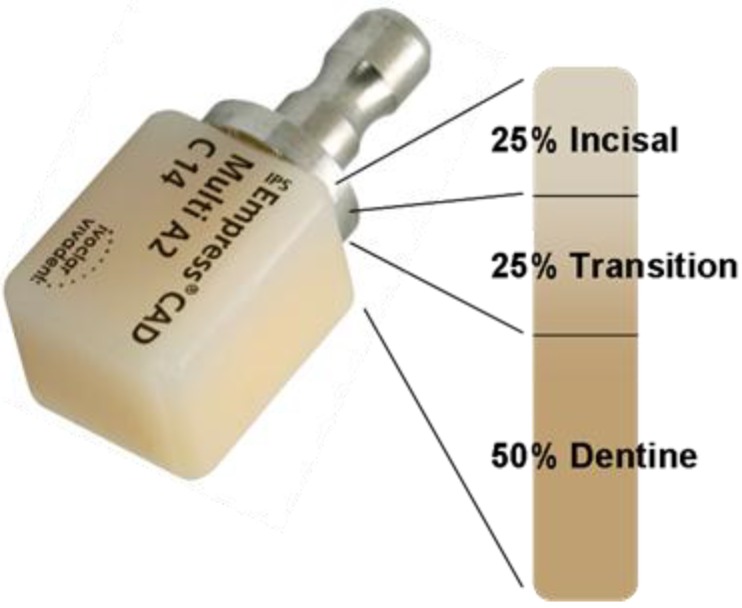
IPS Empress^®^ CAD Multi, Ivoclar Vivadent AG, as multicolor block.

A typical product of leucite based glass-ceramic is IPS Empress^®^ CAD, Ivoclar Vivadent AG. This product has also been developed as multi-colored CAD/CAM blocks, which feature different colors as well as different levels of translucency and brightness [15,16]. This block allows the optical properties of natural teeth to be closely imitated. To avoid the visible transitions between the individual layers (physically called Mach’s bands) for human eyes, these areas are specially built to create an optical illusion. This type of product is made up of a total of four to eight main and intermediate layers. A schematic diagram of the different layers of the leucite glass-ceramic IPS Empress^®^ CAD Multi block is shown in Figure 2.

Due to these highly esthetic properties, this glass-ceramic is mainly used to fabricate anterior crowns as well as inlays and onlays. The entire process from the clinical pre-operative situation (prepared tooth) to the fabrication of the dental restoration with the apparatus Cerec 3 (Sirona, Germany) and ending with the adhesive cementation of the completed restoration takes about two hours.

### 2.2. Type II: Lithium disilicate-based glass-ceramics

In order to extend the indication range of glass-ceramics beyond that of the anterior teeth, a glass-ceramic had to be developed that showed significantly higher strength and fracture toughness compared with the leucite type glass-ceramics. Therefore, a new chemical system, based on a lithium disilicate glass-ceramic was developed to meet this need. Controlled volume nucleation and crystallization allowed a lithium disilicate glass-ceramic to be developed in the SiO_2_-Li_2_O-K_2_O-ZnO-P_2_O_5_-Al_2_O_3_ system. This material demonstrates a significantly higher crystal content (up to 70 vol %) compared with that of leucite glass-ceramics. Due to the high crystal content and the high degree of interlocking crystals, this glass-ceramic exhibits a strength of 350 MPa and a fracture toughness of 2.5 MPa m^1/2^. This material (IPS e.max^®^ Press, Ivoclar Vivadent AG) is suitable for fabricating crowns and frameworks for three-unit bridges using the well-established press technique [17,18]. These products are subsequently coated with a fluoroapatite glass-ceramic in order to imitate the optical properties of natural teeth. The reliability of this material was shown by several *in vitro* and *in vivo* studies [19,20].

**Figure 3:**
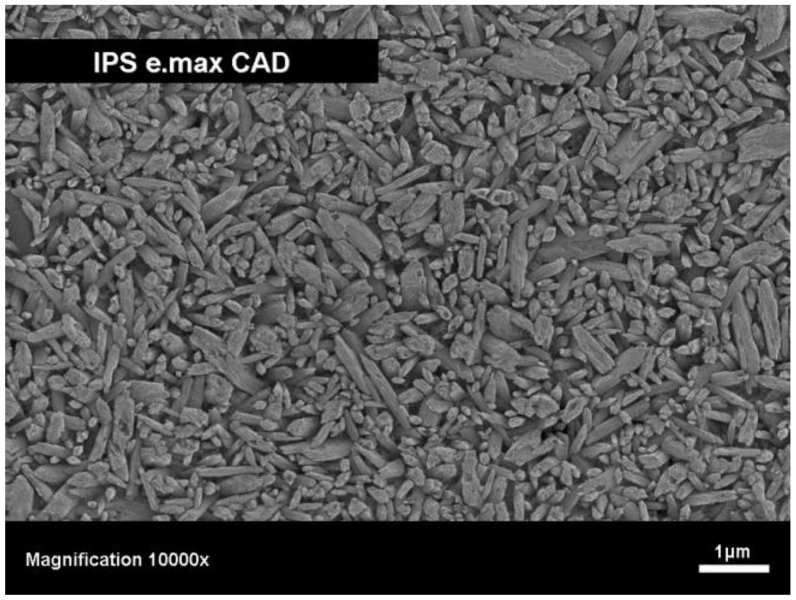
SEM image of the microstructure of a lithium disilicate-type glass-ceramic (IPS e.max^®^ CAD, Ivoclar Vivadent AG), etched with 40% HF vapor for 30 seconds.

Furthermore, a glass-ceramic of lithium disilicate-type needed to be developed for CAD/CAM applications. As lithium disilicate is very difficult to machine with diamond tools by using Cerec 3 (Sirona, Germany) and the base glass is too brittle, other procedures had to be explored in order to allow this glass-ceramic to be machined with CAD/CAM equipment. This challenge was met with the development of an intermediate phase in the SiO_2_-Li_2_O-K_2_O-P_2_O_5_-Al_2_O_3_-ZrO_2_ system [21,22,23,24,25]. In a heat treatment process, lithium metasilicate was precipitated. The glass-ceramic produced in this way shows preferable machining properties. In its intermediate stage the material has a bluish color but exhibits very low chemical durability. However, these properties change significantly during the crystallization process at 850 °C in which the lithium metasilicate is transformed into a durable lithium disilicate glass-ceramic with dental color. Solid state reactions significantly improve the chemical durability of the material and impart the tooth-like optical properties. Table 1 shows the main properties of the investigated lithium disilicate glass-ceramic and Figure 3 shows an SEM image of the interlocking microstructure and the high crystal content.

**Table 1:** Properties of a leucite-type glass-ceramic IPS Empress^®^ CAD and a lithium disilicate-type glass-ceramic IPS e.max^®^ CAD, Ivoclar Vivadent AG.

Properties		IPS Empress^®^ CAD	IPS e.max^®^ CAD
Biaxial flexural strength	MPa	160	300–420
Fracture toughness, K_IC_	MPa m^1/2^	1.3	2.0–2.5
Hardness	MPa	6200	5700–5900
Elastic modulus	GPa	62	90–100
CTE_(100–500 °C)_	10^-6^ K^-1^	17.0–18.0	10.2–10.7
Chemical durability (weigth loss in 4% acidic acid)	μg cm^-2^	25	30–50

### 2.3. Type III: Yttrium-stabilized zirconium oxide-based ceramic

Yttrium-stabilized zirconium oxide as polycrystalline sintered ceramic is applied in dentistry specially as crown and bridge frameworks [26,27,28,29]. Apart from being used to produce crown copings and bridge frameworks, this material is suitable for fabricating posts [30], abutments [31] and implants. Yttrium-stabilized zirconium oxide ceramics are characterized by high strength and fracture toughness. The biaxial strength measures between 900 and 1200 MPa and the fracture toughness as K_IC_ value measured according to the dental ISO Standard 6872:2008 by the SEVNB (single-edge V-notched-beam) method is 4–5 MPa m^1/2^. The reinforcement mechanism which is based on the stress-induced phase transformation of a tetragonal to a monocline crystal phase has been examined in various research projects [32,33,34,35,36].

**Figure 4:**
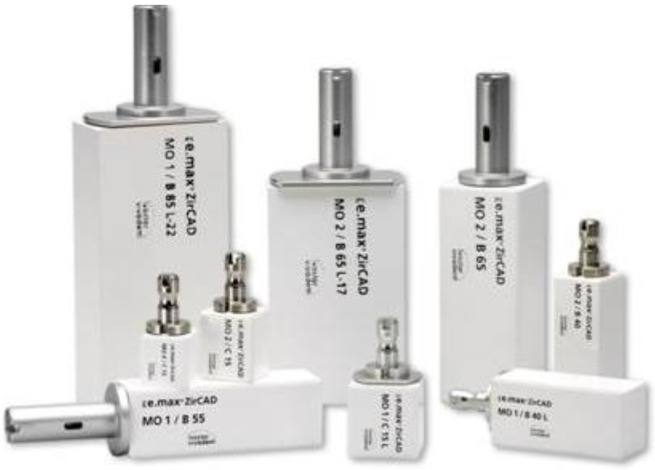
Image of the presintered product IPS e.max^®^ ZirCAD, Ivoclar Vivadent AG, with the metal holder for CAD/CAM-technology.

**Figure 5:**
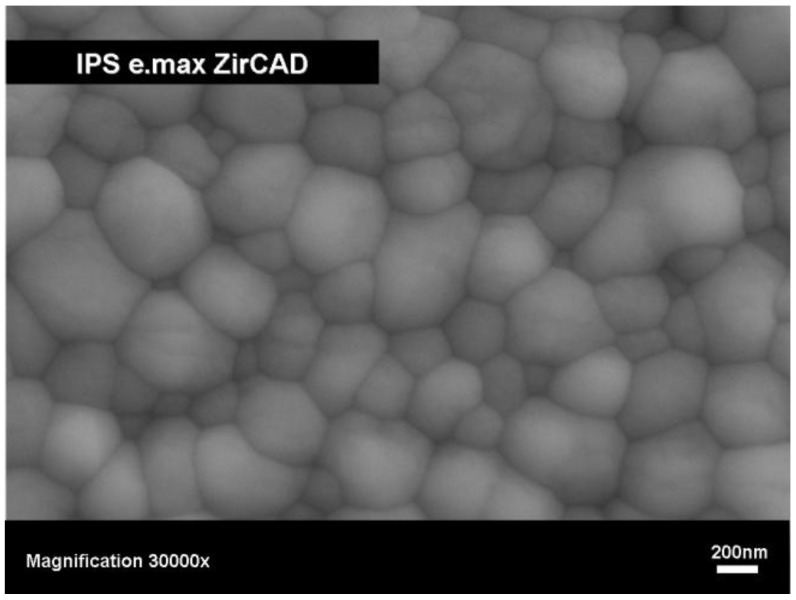
SEM image of the microstructure of IPS e.max^®^ ZirCAD, Ivoclar Vivadent AG, after final densification, polished surface after thermal etched 1420 °C for 15 minutes.

Two different processes [29] are available for fabricating dental restorations using zirconium oxide: (a) machining of dense ceramics and (b) machining of presintered ceramics. The first method involves milling densely sintered or even hot isostatic pressed (HIP) zirconium oxide. This process is very time-consuming and the corresponding machining equipment is a large, heavy multi axis machining apparatus. The second method is available in which zirconium oxide is milled in a porous state using a small desktop machining apparatus, like Cerec 3 (Sirona, Germany). In fact, this way of producing ZrO_2_-based restorations has already become firmly established in dentistry. The porosity, hardness and strength of the material are coordinated to optimize the relationship between the machining time, the wear of the tools and the final properties of the zirconium oxide. In Table 2, the properties of these porous ZrO_2_ blanks are shown. The processing of these porous blanks (Figure 4) has to be very accurate, because the homogeneity of the density and the pore size distribution influences the properties of the final product. After the restoration has been created using the CAD/CAM equipment, it has to be densely sintered. This is done in a high-temperature furnace at temperatures between 1400 and 1500 °C. In Figure 5 the final microstructure of the zirconia material is demonstrated and the properties of this product are shown in Table 2. The ZrO_2_ framework is subsequently covered with a fluoroapatite glass-ceramic using the build-up or press technique.

**Table 2:** Properties of IPS e.max^®^ ZirCAD, Ivoclar Vivadent AG; in the presintered and final sintered state, including chemical composition.

Presintered ZrO_2_			Final dense sintered ZrO_2_		
Properties			Properties		
Density	g cm^-3^	3.09–3.21	Density	g cm^-3^	>6.0
Porosity	%	47.3–49.3	Porosity	%	<0.5
Biaxial flexural strength	MPa	50–90	Biaxial flexural strength	MPa	>900
ZrO_2_	wt %	87.0–95.0	Fracture toughness, K_IC_	MPa m^1/2^	5.5
Y_2_O_3_	wt %	4.0–6.0	Hardness HV10	MPa	13000
HfO_2_	wt %	1.0–5.0	CTE_(100–400°C)_	10^-6^ K^-1^	10.75
Al_2_O_3_	wt %	0.1–1.0	CTE_(100–500°C)_	10^-6^ K^-1^	10.8

In recent years, colored ZrO_2_ ceramics have been developed for dental applications. Different procedures of coloring ZrO_2_ frameworks are available. One possibility is the infiltration of porous ZrO_2_ ceramic with special color solutions. The dental color is visible after the infiltrated ZrO_2_ frameworks have been densely sintered.

A different possibility has opened up with the introduction of colored ZrO_2_ blocks [28]. Restorations can now be fabricated without requiring infiltration. As a result, one working step is eliminated for the dental laboratory.

## 3. Machining Systems using CAD/CAM Technology

**Figure 6:**
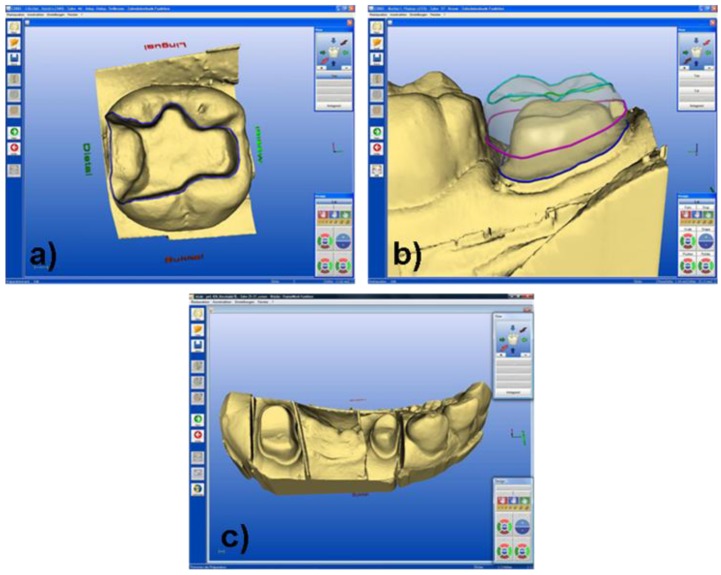
**(a)** Digital 3D model of the prepared tooth, (tooth #46), corresponding to the clinical case of Figure 8. **(b)** Digital 3D model of the prepared tooth, (tooth #37), corresponding to the clinical case of Figure 9. **(c)** Digital 3D model of the prepared tooth, (tooth #24–#27), corresponding to the clinical case of Figure 10.

Most applied machining systems using CAD/CAM methods include the products of the following companies Procera^®^ (Nobel Biocare, Göteborg, Sweden), DCS Precimill (DCS Dental AG, Allschwil, Switzerland), LAVA^TM^ (3M Espe Dental AG, Seefeld, Germany) KaVoEverest^®^ (KaVO EWL, Leutkirch, Germany), ZENOTECH^TM^ (Wieland Dental und Technik, Pforzheim, Germany), E4D (D4D, Richardson, Texas, USA), Cercon^®^ (DeguDent GmbH, Hanau, Germany) and Decim (Decim AB; Skellefteå, Sweden). These systems carry out machining processes with diamond tools. CEREC 3 (Sirona, Germany) is one of the most widely used dental CAD/CAM systems [37]. It is designed to machine glass-ceramic and ceramic restorations. The system is composed of three different modules, which are required in the fabrication of precision restorations. With this system the dentist can scan the prepared teeth with an intraoral camera, which transmits the recorded information directly to the computer and transforms it into a digital image (Figure 6a and Figure 6b). Alternatively, the dentist can take an impression of the patient’s dentition and send this to the dental laboratory, where it is used to create a plaster model. Subsequently, the surface of the model is optically recorded to produce a digital image (Figure 6c).

**Figure 7:**
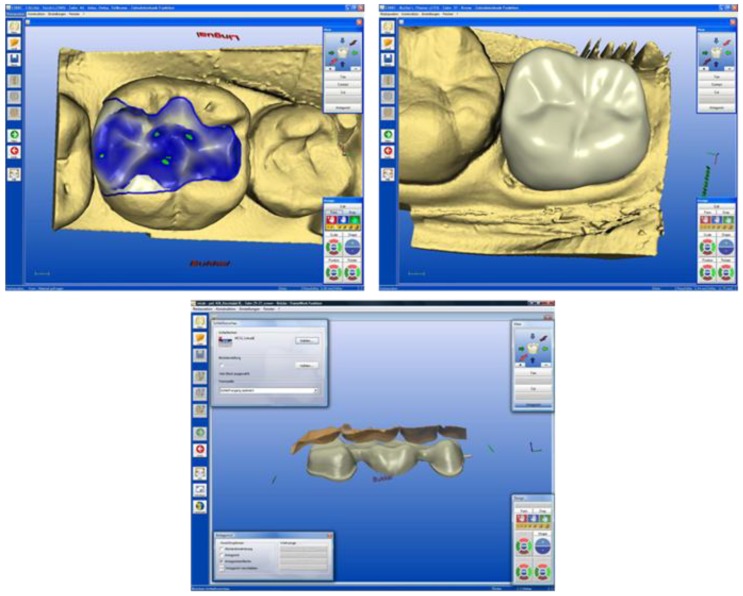
**(a)** Digital 3D model of the virtual inlay, (tooth #46), corresponding to the clinical case of Figure 8. **(b)** Digital 3D model of the virtual crown, (tooth #37), corresponding to the clinical case of Figure 9. **(c)** Digital 3D model of the virtual bridge, (tooth #24–#27), corresponding to the clinical case of Figure 10.

Subsequently, the dentist (chair-side) or the dental technician (lab-side) can design the restoration using the CAD software (Figure 7).

The data of the virtual model is used to mill a ceramic block to the desired shape with diamond tools. After the machining process, the appearance of the restoration is adjusted by the dentist with special “shade” and “effect” materials. Then, the restoration is ready for placement in the patient’s mouth. Zirconium oxide-based restorations that are fabricated in the dental laboratory have to be densely sintered to harden them. As the optical properties of zirconium oxide do not correspond to those of natural teeth, a fluoroapatite-based glass-ceramic has to be either built up on or pressed to the substructure. This additional and very time-consuming step can only be carried out by a dental technician in the dental laboratory.

## 4. Clinical Application

**Figure 8:**
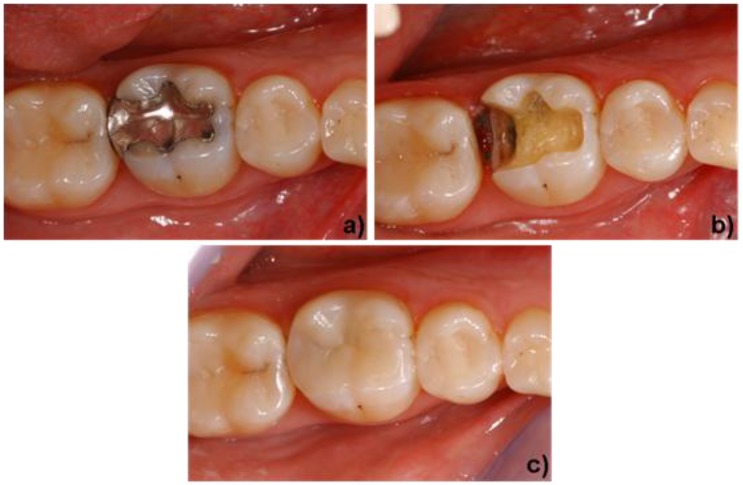
Application of a leucite-based glass-ceramic. a) initial situation (tooth #46), damaged occlusal and distal amalgam filling. b) minimally invasive preparation of the molar for an inlay restoration (IPS Empress^®^ CAD). c) final clinical situation after adhesive luting and polishing of the inlay. Dentist: A. Peschke (Ivoclar Vivadent AG).

The materials that are used to create a functional, long-lasting and esthetic restoration are dictated by the clinical pre-operative situation. The three types of materials that are commercially available today are discussed in Section 2. Highly esthetic glass-ceramics exhibiting strength values of 200-400 MPa are mainly used to restore anterior teeth [29]. Figure 8 shows a clinical case in which leucite glass-ceramics were indicated. The material is well known and often used for CAD/CAM technology [38,39,40,41]. In this case, an amalgam filling was replaced with an inlay that was fabricated with CAD/CAM methods. The tooth was prepared and the inlay created in one dentist appointment, in other words, chair-side. Due to the low strength of leucite glass-ceramics, the application of these materials is restricted to the fabrication of inlays, onlays and anterior crowns.

**Figure 9:**
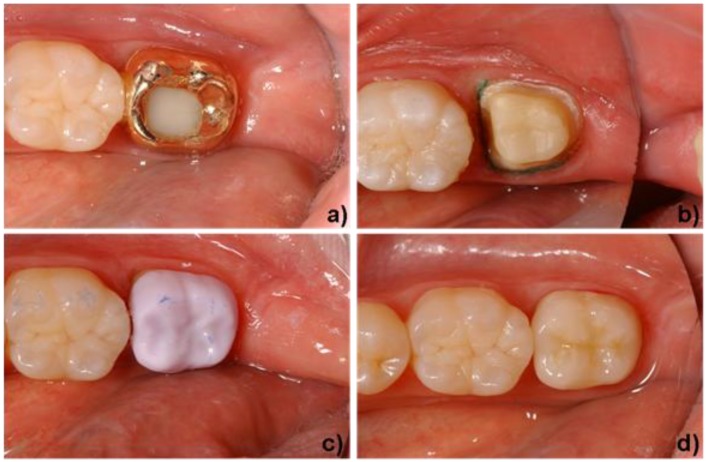
Application of a lithium disilicate-based glass-ceramic. a) initial situation (tooth #37), worn gold crown. b) preparation of the molar for a full contour crown (IPS e.max^®^ CAD). c) try-in of a full contour crown in its lithium metasilicate state. d) final clinical situation after the crystallization step (stained and glazed) and adhesive cementation of the crown. Dentist: A. Peschke (Ivoclar Vivadent AG).

The indication range of glass-ceramics has been considerably enlarged with the advent of lithium disilicate glass-ceramics. Figure 9 shows this material being used to create a full-anatomic crown for a posterior tooth. The worn gold crown, which had to be replaced, is shown in Figure 9a. The crown was removed and the remaining tooth structure was prepared to receive the new restoration. The dentist used an intraoral camera to capture a digital image of the prepared tooth. On the basis of this image, a virtual model of the final restoration was created with CAD software. Subsequently, the restoration was milled from a lithium metasilicate block. Figure 9c shows the full-anatomic crown in a partially crystallized state (lithium metasilicate) during try-in. Next, the dentist customized the crown with characterization stains and a glaze. The lithium metasilicate material was heat treated to transform it into its final high-strength lithium disilicate state. After the firing process, the restoration exhibited a natural tooth-color and was adhesively cemented. The result is shown in Figure 9d.

**Figure 10:**
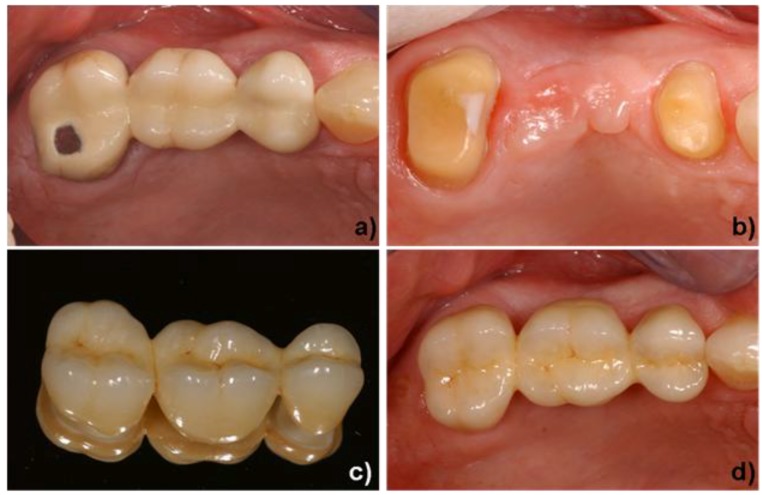
Application of a veneered zirconium oxide ceramic. a) initial situation (tooth #24–27), damaged PFM bridge. b) preparation of tooth #24 and #27 for a veneered zirconium oxide 3-unit bridge restoration (IPS e.max^®^ ZirCAD), teeth #25 and #26 have been extracted. c) all-ceramic bridge on a mirror; the high-strength, white IPS e.max^®^ ZirCAD framework is veneered by a fluoroapatite glass-ceramic (IPS e.max^®^ Ceram). d) final clinical situation after cementation with a glass ionomer cement. Dentist: A. Peschke; Dental technician: F. Perkon (both Ivoclar Vivadent AG).

In the posterior region, the use of glass-ceramics is restricted to single-tooth restorations (inlays, onlays and crowns), because of the high forces exerted in this part of the mouth. Consequently, long-span bridges in this region are usually fabricated with high-strength and tough oxide ceramics (ZrO_2_). Subsequently, glass-ceramics are either built up on or pressed to these oxide ceramic substructures to imitate the optical and tribological properties of the natural dentine. Figure 10 shows a clinical case in which a damaged porcelain fused to metal (PFM) bridge had to be replaced. The dentist removed the old restoration and prepared the two remaining teeth to receive the new bridge. In this case, tooth #25 and #26 were missing. Because of restricted space, these two teeth were replaced by a single pontic. As the bridge had to be fabricated in the dental lab, the dentist made an impression (negative) of the remaining teeth. This impression was sent to the dental lab, where a plaster model (positive) was produced and the surface of the model was optically recorded (Figure 6c). With the help of the CAD software, the lab technician designed the bridge framework (Figure 7c), which was subsequently milled from a presintered zirconium block. When the ZrO_2_ bridge was designed, the shrinkage factor of the material was taken into consideration automatically. Consequently, the dimensions of the restoration after milling were approximately 20% larger than those of the final restoration. In order to obtain the desired properties of the ZrO_2_ material, the framework had to be densely sintered at temperatures between 1400 and 1500 °C. Since ZrO_2_ is a very white opaque and hard material, its color, brightness and translucency and its wear characteristics are not tooth-like. Therefore, a fluoroapatite glass-ceramic was built up onto the bridge framework. The dental technician’s finished piece of work is shown in Figure 10c. The restoration was placed with glass ionomer cement. The final restoration, shown in Figure 10d, demonstrates tooth-like colors due to the additional coating with glass-ceramic. Furthermore, the material properties of the ZrO_2_ substructure ensure that it functions well: several *in vitro* and short-term *in-vivo* studies showed good survival rates [42,43,44,45,46,47].

## References

[1] Kirsten H. (1929). Die Jacketkrone.

[2] Southan D.E., Mc Lean J.W. (1984). Die Porzellan-Jacketkrone. Dental-Keramik Vorträge und Diskussionen.

[3] Hoffmann-Axelthelm W. (1974). Geschichte der Zahnheilkunde.

[4] Gehre G., Eichner K., Kappert H.F. (2005). Zahnärztliche Werkstoffe und ihre Verarbeitung. Band 1. Grundlagen und Verarbeitung.

[5] Kerschbaum T. (1986). Überlebenszeiten von Kronen und Brückenzahnersatz heute. Zahnärztl. Mitt..

[6] Kerschbaum T., Erpenstein H. (1997). Galvano-keramische Einzelkronen haben sich klinisch bewährt. Zahnärztl. Mitt..

[7] Weinstein M., Katz S., Weinstein A.B. (1962). Fused Procelain-To-Metal Teeth. U.S. Patent.

[8] McLean J.W., Dickson G., Cassels J.M. (1972). Dental Porcelains. Dental Materials Research.

[9] Wohlwend A., Schärer P. (1990). Die empress-technik—ein neues Verfahren zur Herstellung von vollkeramischen Kronen, Inlays und Facetten. Quintessenz Zahntech.

[10] Höland W., Frank M., Haller B., Bischoff H. (1993). IPS Empress Glaskeramik. Metallfreie Restaurationen aus Presskeramik.

[11] Mörmann W.H., Jans H., Brandestini M., Ferru A., Lutz F. (1986). Computer machined adhesive porcelain inlays: margin adaptation after fatigue stress. J. Dent. Res..

[12] Duret F. (1988). CAD/CAM in dentistry. J. Am. Dent Assoc..

[13] Rekow E.D. (1988). Prostheses by computer. N.Y. State Dental J..

[14] Höland W., Frank M., Rheinberger V.M. (1995). Surface Crystallization of Leucite in Glass. J. Non-Cryst. Solids.

[15] Schweiger M. Materials Properties of IPS Empress. Presented at Scientific Meeting.

[16] Bühler P., Völkel T. (2006).

[17] Schweiger M., Höland W., Frank M., Drescher H., Rheinberger V.M. (1999). IPS Empress 2, a new pressable high strength glass-ceramic for esthetic all ceramic restoration. Quint. Dent. Technol..

[18] Höland W., Schweiger M., Frank M., Rheinberger V.M. (2000). A comparison of the microstructure and properties of the IPS Empress 2 and the IPS Empress glass-ceramic. J. Biomed. Mater. Res. Part B.

[19] Sorensen J.A., Cruz M., Mito W.T., Merrideth H., Raffeiner O. (1999). Empress 2 all-ceramic bridge clinical trails. IADR Abstract 902, Symposia Behavioral Sciences & Health Services Research. J. Dent. Res..

[20] Pospiech P., Rountree P., Unsöld F., Rammelsberg P. (1999). *In vitro*-investigations on the fracture strength of all-ceramic posterior bridges of Empress II. J. Dent. Res..

[21] Apel E., van’t Hoen C., Rheinberger V., Höland W. (2007). Influence of ZrO_2_ on the crystallization and properties of lithium disilicate glass-ceramics derived from a multi-component system. J. Eur. Ceram. Soc..

[22] Höland W., Rheinberger V., Apel E., van’t Hoen C. (2007). Principles and Phenomena of bioengineering with glass-ceramics for dental restoration. J. Eur. Ceram. Soc..

[23] Höland W., Apel E., van’t Hoen C., Rheinberger V. (2006). Studies of crystal phase formations in high-strength lithium disilicate glass-ceramics. J. Non-Cryst. Solids.

[24] Höland W., Rheinberger V., van’t Hoen C. (2005). P_2_O_5_ as an effective nucleating agent of lithium disilicate glass-ceramics. J. Inorg. Phophorus Chem..

[25] Ritzberger C., Rheinberger V., Höland W., Apel E. Hochfeste Glaskeramik, 80. Presented at Glastechnische Tagung & 8th International Conference “Advances in Fusion and Processing of Glass”.

[26] Schweiger M. (2004). Zirkonoxid—hochfeste und bruchzähe Strukturkeramik. Ästhetische Zahnmedizin.

[27] Rothbrust F. (2006). IPS e.max ZirCAD.

[28] Höland W., Rheinberger V., Apel E., Ritzberger C., Rothbrust F., Kappert H., Krumeich F., Nesper R. (2009). Future perspectives of biomaterials for dental restoration. J. Eur. Ceram. Soc..

[29] Höland W., Schweiger M., Watzke R., Peschke A., Kappert H.F. (2008). Ceramics as biomaterials for dental restoration. Expert Rev. Med. Devices.

[30] Sorensen M., Mito W.T. (1998). Rational and clinical technique for esthetic restorations of endodontically treated teeth with the Cosmo Post and IPS Empress Post system. Quint. Dent. Technol..

[31] Wohlwend A., Studer S., Schärer P. (1996). Das Zirkondioxidabutment - ein neues vollkeramisches Konzept zur ästhetischen Verbesserung der Suprastrukturen in der Implantologie. Quintessenz Zahntech..

[32] Rühle M., Evans A.G. (1989). High toughness ceramics and ceramic composites. Prog. Mat. Sci..

[33] Deville S., Guénin G., Chevalier J. (2004). Martensitic transformation in zirconia part I. Nanometer scale prediction and measurement of transformation induced relief. Acta Mater..

[34] Deville S., Guénin G., Chevalier J. (2004). Martensitic transformation in zirconia part II. Martensite growth. Acta Mater..

[35] Lange F.F. (1982). Transformation Toughening. J. Mat. Sci..

[36] Lange F.F. (1986). Transformation-Toughened ZrO_2_: Correlations between grain size control and composition in the system ZrO_2_-Y_2_O_3_. J. Am. Ceram. Soc..

[37] Mörmann W.H. (2006). State of the Art of CAD/CAM Restorations.

[38] Mörmann W.H., Brandestini M. (1989). Chairside computer-aided direct ceramic inlays. Quintessence Int..

[39] Mehl A., Gloger W., Hickel R. (2000). Fully anatomic CAD/CAM-fabrication of tooth restorations with a new precise 3D-scanning system. J. Dent. Res..

[40] Mörmann W.H. (2006). 20 Jahre keramische CEREC CAD/CAM restaurationen. technischer stand und klinische bewährung. Zahnärztl. Mit..

[41] Reich S., Wichmann M. (2004). Unterschiede zwischen den CEREC-3D-Software-Versionen 1000 und 15000. Int. J. Comp. Dent..

[42] Sailer I., Pjetursson B.E., Zwahlen M., Hämmerle C.H. (2007). A systematic review of the survival and complication rates of all-ceramic and metal-ceramic reconstructions after an observation period of at least 3 Years. Part II: fixed dental prostheses. Clin. Oral Implants Res..

[43] Att W., Grigoriadou M., Strub J.R. (2007). ZrO_2_ three unit fixed partial dentures: comparison of failure load before and after exposure to a mastication simulator. J. Oral Rehabil..

[44] Vult von Steyern P., Ebberson S., Holmgren J., Haag P., Nilner K. (2006). Fracture strength of two oxide ceramic crown systems after cyclic pre-loading and thermocycling. J. Oral Rehabil..

[45] Tinschert J., Natt G., Mautsch W., Augthun M., Spiekermann H. (2001). Fracture resistance of lithium disilicate-, alumina- and zirconia-based three unit fixed partial dentures: a laboratory study. Int. J. Prosthodont..

[46] Blatz M.B. (2002). Long therm clinical success of all-ceramic posterior restorations. Quintessence Int..

[47] Sailer I., Feher A., Filser F., Gauckler L.J., Luthy H., Hämmerle C.H. (2007). Five-year clinical results of zirconia frameworks for posterior fixed partial dentures. Int. J. Prosthodont..

